# Supplementary material for: Complexome profiling of the Chlamydomonas psb28 mutant reveals TEF5 as an early PSII assembly factor
Source: Plant Cell. 2025 Mar 17;37(6):koaf055. doi: 10.1093/plcell/koaf055 (PMC12164590; doi:10.1093/plcell/koaf055)
Supplement: koaf055_Supplementary_Data [file koaf055_supplementary_data.zip › psb28_interactive.zip!/plots/intensity/qualityPlots/peptides/_Cre08.g358540.t1.1.html]

### Cre08.g358540.t1.1

Trivial name:   
SeqCoverage [median]: 37.70  
Mass: 27.17 kDa  
  
 

| Sequence | MissedCleavage | Mass [Da] | wtIntensity | muIntensity | Score |
| --- | --- | --- | --- | --- | --- |
| AVYDYSDEK | 0 | 1088.47 | 5098.37 | 4695.13 | 124.98 |
| ELQAYILR | 0 | 1004.57 | 3824.66 | 17993.62 | 155.88 |
| GAQLTQEELLGEPVAGGK | 0 | 1795.93 | 15777.43 | 25075.20 | 277.82 |
| GAQLTQEELLGEPVAGGKGK | 1 | 1981.05 | 1881.07 | 0.00 | 39.24 |
| GATPYGEGVK | 0 | 977.48 | 6387.70 | 9549.69 | 86.67 |
| GLFVNYR | 0 | 867.46 | 2892.73 | 8597.85 | 105.42 |
| IEMRALDNFR | 1 | 1263.64 | 752.59 | 509.30 | 45.14 |
| NEQLDAAYQEVK | 0 | 1406.67 | 0.00 | 1253.05 | 179.19 |
| RPVVEDAPTPK | 1 | 1207.66 | 5207.05 | 7578.20 | 107.34 |
| TTAPWKNEQLDAAYQEVK | 1 | 2091.03 | 2877.73 | 1583.91 | 120.63 |
|  |  |  | 44699.3 | 76835.9 |  |
